# Case report: Drainage tube penetrating anastomosis as a rare cause for long-term nonunion of esophagogastric anastomosis in neck

**DOI:** 10.3389/fsurg.2023.1140839

**Published:** 2023-02-22

**Authors:** Yaochen Huang, Xiangning Fu, Shengling Fu

**Affiliations:** Department of Thoracic Surgery, Tongji Hospital, Tongji Medical College, Huazhong University of Science and Technology, Wuhan, China

**Keywords:** esophageal cancer, McKeown esophagectomy, anastomotic leakage, cervical drainage, case report

## Abstract

Anastomotic leakage is a life-threatening complication for esophageal cancer patients who received McKeown esophagectomy. Cervical drainage tube penetrating anastomosis is a rare but noteworthy cause of long-term nonunion of esophagogastric anastomosis. Here we reported two cases of esophageal cancer patients who received McKeown esophagectomy. The first case acquired the anastomotic leakage on postoperative day (POD) 7, and lasted for 56 days. The cervical drainage tube was removed at POD 38, and the leakage healed in 25 days. The second case acquired the anastomotic leakage on POD 8 and lasted for 95 days. The cervical drainage tube was removed at POD 57, and the leakage healed in 46 days. The two cases demonstrated the duration-prolonging effect of drainage tube penetrating anastomosis, which should not be overlooked in clinical practice. We suggested paying attention to the duration of leakage, the drainage fluids amounts and characteristics, and the imaging manifestations to help diagnose. If the cervical drainage tube penetrated the anastomosis, the tube should be eliminated as soon.

## Introduction

Esophageal cancer has become the tenth most common cancer worldwide and the fifth most common cancer in China with an incidence of more than three hundred thousand new cases annually (1, 2). Esophagectomy and esophagogastric anastomosis for digestive tract reconstruction is an effective approach to cure localized esophageal cancer and prolong the life of patients (3, 4). One of the frequently-used approaches for surgery is McKeown esophagectomy (5).

Anastomotic leakage is a common but life-threatening complication reported in 8%–35% of patients receiving esophagectomy (6, 7). We noticed a rare but notable factor for delaying the healing of anastomotic leakage, which is the cervical wound drainage tube penetrating the anastomotic stoma among patients who underwent McKeown esophagectomy. Here, we report two cases of patients with the situation mentioned above.

## Case description

### Case 1

**Figure 1:**
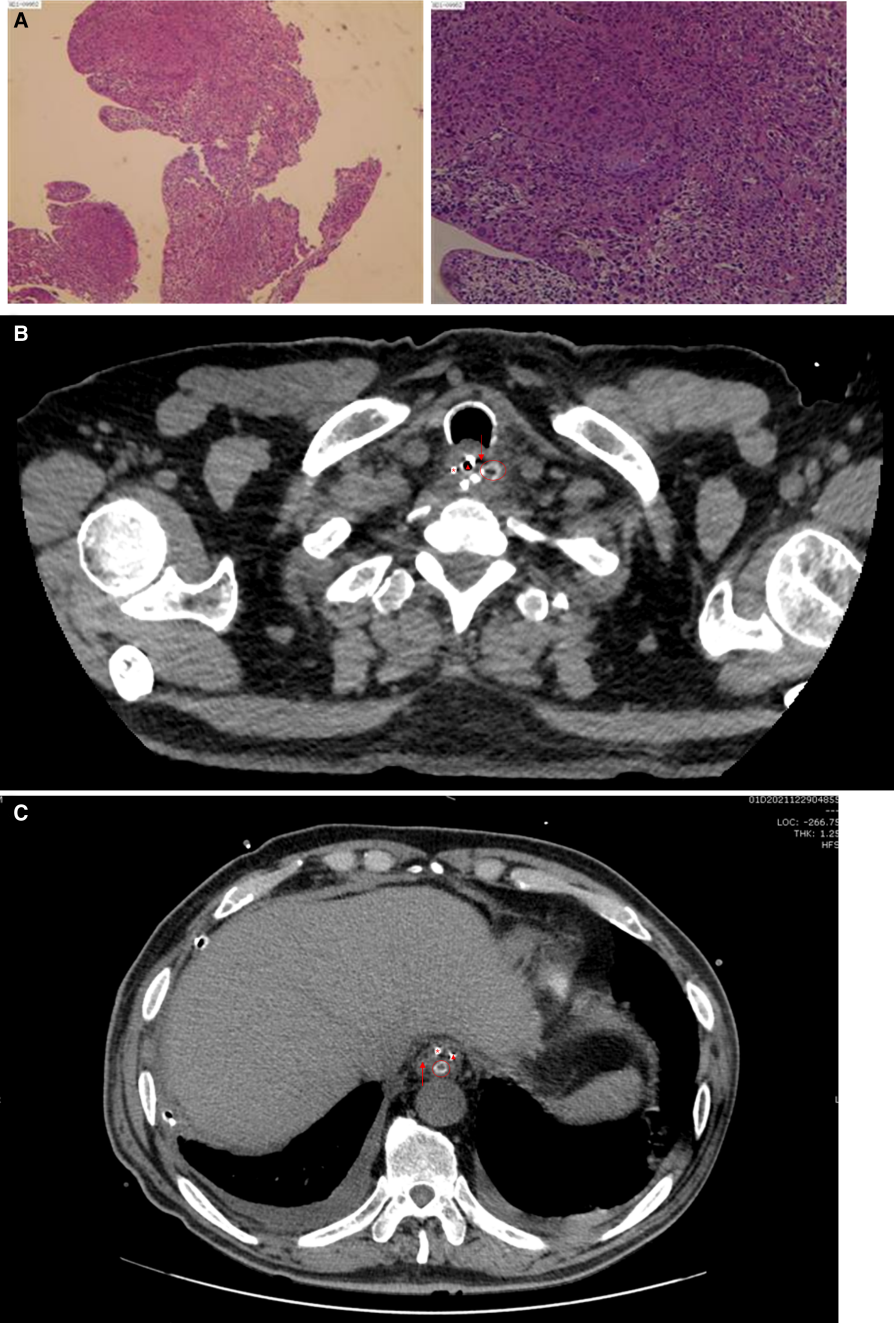
(**A**) Preoperative pathological pictures of the patient; (**B**) CT image on POD 7. Images showed that the drainage tube was located outside the esophageal cavity; (**C**) CT image on POD 37 showed that the cervical drainage tube went deep into the stomach. ○, cervical drainage tube; ▴, gastric tube; *, duodenal nutrient tube; ↓, esophageal cavity; ↑, stomach.

A 67-year-old man with a history of hypertension, type 2 diabetes mellitus, hyperlipidemia, atrophic gastritis, and duodenal ulcer was referred to the clinic due to the feeling of aggravating dysphagia for 2 months. The gastroscope revealed a neoplasm 23–30 cm away from the upper incisors, and the biopsy indicated squamous cell carcinoma (SCC) (Figure 1A). Along with the endoscopy ultrasound (EUS) findings, the patient was preoperatively diagnosed with cT1aN1M0 Stage I esophageal cancer according to the AJCC/UICC cancer staging manual (8th edition) (8). The minimally invasive Mckeown esophagectomy was performed. A cervical drainage tube was positioned routinely. The post-operation pathological diagnosis was SCC, moderately differentiated, and lamina propria invasion locally, which indicated the diagnosis of pT1aN1M0 Stage I esophageal cancer.

Anastomotic leakage was reached on the seventh postoperative day (POD 7), and the patient presented with cervical hemorrhage on POD 13. Emergency surgery of cervical exploration, hemostasis, and drainage was performed. We noticed that since POD 20 (the eighth day after the second surgery), the volume of cervical drainage increased while the volume of gastric tube drainage decreased. On POD 25, the volume of cervical drainage reached the maximum (800 ml), while the gastric tube drainage reached the minimal (20 ml), and the cervical drainage fluid turned to brown mucus from hematic liquid. Hence, we suspect that the cervical drainage tube penetrated the anastomotic stoma. After reviewing the radiographic images retrospectively, we noticed that the drainage tube was located outside the esophageal cavity when the leakage emerged on POD 7 (Figure 1B) but the CT on POD 37 showed that the cervical drainage tube went deep into the stomach (Figure 1C), a video of continuous slices of CT was available (Supplementary Video S1), which explained why the neck has a lot of drainages. Thus, we removed the cervical drainage tube on POD 38, continued conservative management, and conducted CT or Iodine hydrography of esophagus for monitoring routinely. On POD 62, Iodine hydrography of esophagus showed that the anastomosis had healed. The total postoperative duration of disease was 62 days and the disease course of anastomotic leakage lasted for 56 days. After removing the cervical drainage tube, the leakage healed in 25 days.

### Case 2

**Figure 2:**
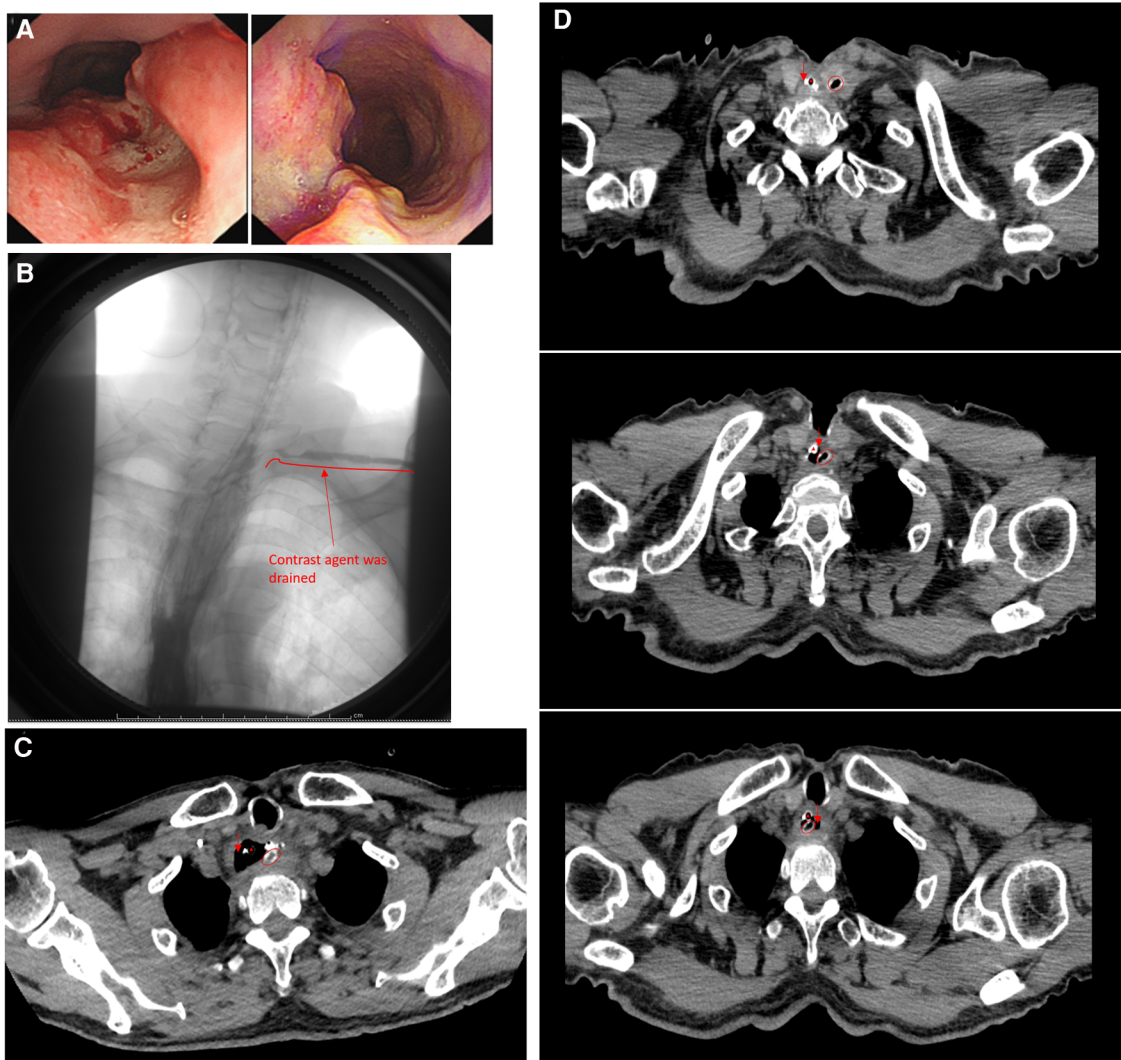
(**A**) Preoperative gastroscopic pictures of the patient; (**B**) esophagography on POD 57, contrast agent was drained through the cervical drainage tube; (**C**) CT on POD 8, the cervical drainage clung to the esophageal wall but not penetrate into; (**D**) three slices of CT images on POD 12 showed the extend of the cervical drainage tube from the cervical wound to the esophageal cavity. ○, cervical drainage tube; ▴, gastric tube; ↓, esophageal cavity.

A 72-year-old man with a history of laryngeal carcinoma which has been surgically removed 6 years ago was referred to the clinic due to subxiphoid pain for 7 months accompanied by aggravating dysphagia for 6 months. The gastroscope revealed a neoplasm 27–30 cm away from the upper incisors, and the biopsy indicated SCC (Figure 2A). Along with the EUS findings, the patient was preoperatively diagnosed with cT2N2M0 Stage III esophageal cancer. The patient refused to receive neoadjuvant therapy and requested surgery. The minimally invasive Mckeown esophagectomy was performed. A cervical drainage tube was positioned routinely. The post-operation pathological diagnosis was SCC, poorly differentiated and adventitia invasion, one left laryngeal gyrus lymph node, and three lesser curvature of stomach lymph nodes metastasis, which indicated the diagnosis of pT3N2M0 Stage IIIB esophageal cancer. The patient was referred to the department of oncology for further chemotherapy postoperatively.

On POD 8, neck exudation was found and anastomotic leakage was considered.

After a period of conservative management, the esophagography on POD 57 showed that the contrast agent was drained through the cervical drainage tube, indicating the leakage failed to heal (Figure 2B). A retrospective analysis of chest CT revealed that the neck drainage tube did not penetrate the esophageal lumen on POD 8 but was close to the esophageal wall (Figure 2C), which made it possible for the neck drainage tube to penetrate the esophageal lumen, and the neck drainage tube had penetrated the esophageal lumen since POD 12 (Figure 2D). Considering the cervical drainage tube penetrated the anastomotic stoma, the tube was removed immediately (POD 57). Since the patient refused to receive endoscopic intervention, conservative management continued. On POD 102, esophagography showed that the anastomotic stoma was leak-free. The total postoperative disease course was 102 days and the anastomotic leakage lasted for 95 days. The leakage healed 46 days after removing the cervical drainage tube.

**Figure 3:**
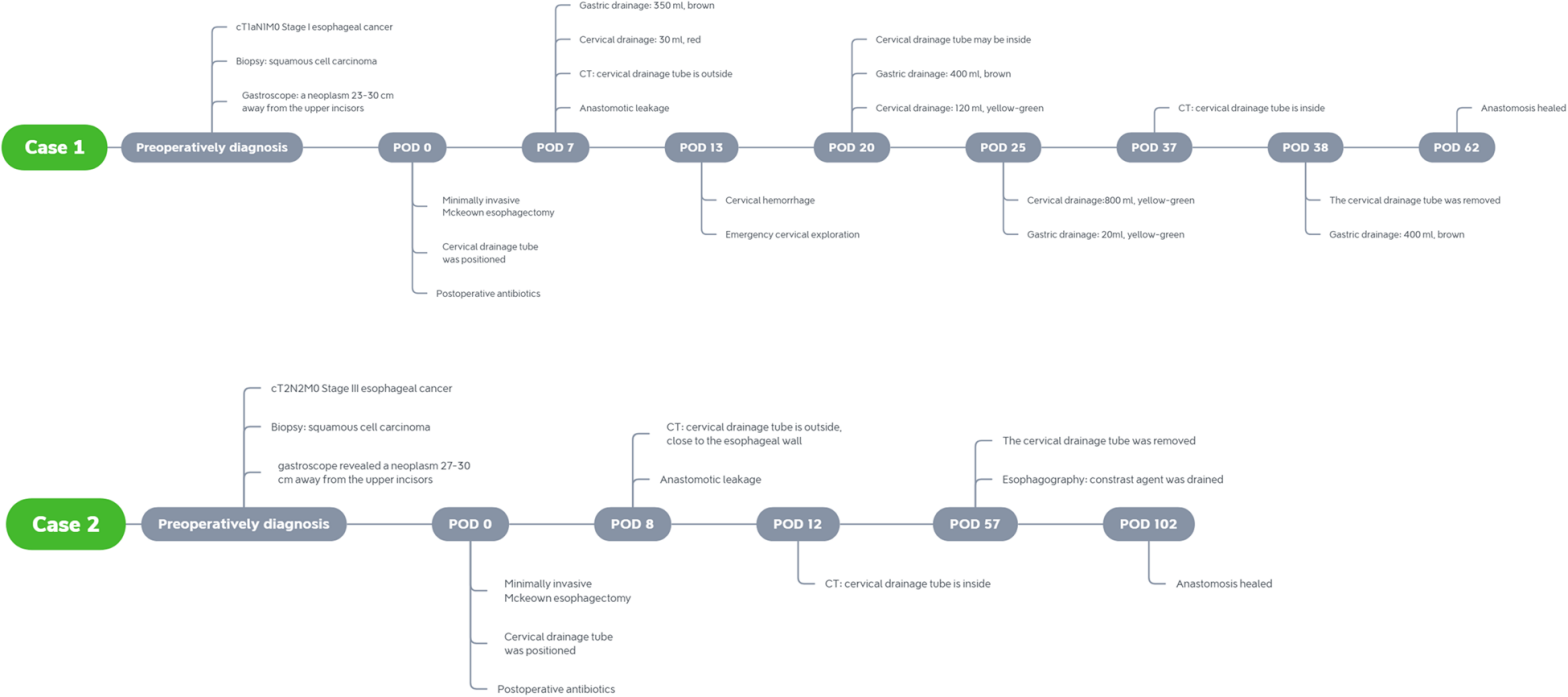
Timelines for the presented cases indicating important events including diagnosis, detailed preoperative baseline data, auxiliary treatment, surgical plan, and postoperative management during the perioperative period.

A timeline of each case indicating important events including diagnosis, detailed preoperative baseline data, auxiliary treatment, surgical plan, and postoperative management during the perioperative period is shown in Figure 3.

## Discussion

Anastomotic leakage is one of the most severe complications of esophagectomy and esophagogastric anastomosis with a mortality rate between 7.2% and 35%, affecting the prognosis adversely (9).

Cervical wound drainage is widely used in many clinical centers. It can not only minimize the dead space after the dissection but also offer information about hemorrhage, chyle leak, or anastomotic leakage, and enable clinicians to start treatment rapidly (10). The usual practice of cervical drain is placing a silicone or polyurethane foam drainage tube posterior to the anastomosis (10, 11). The size of the drainage tube ranges from 10 to 24 Ch/Fr according to physicians’ choices. Negative pressure drainage can be employed for a more efficient drain through a negative pressure balloon (12).

However, due to the routine use of cervical wound drainage after McKeown esophagectomy, the complications caused or aggravated by drainage tubes become noteworthy. The neck is a flexible structure and cervical movement is highly frequent. Plenty of basic life activities require the movement of the neck. Because of the flexibility of the neck, the drainage tube can be easily shifted by muscle contraction, esophageal peristalsis, and vocal fold vibration, causing or aggravating postoperative complications such as inadequate drainage, hemorrhage, infection, and under a rare situation, the tube may penetrate the anastomotic stoma, resulting in protracting the healing of anastomotic leakage as the cases presented above. Anastomotic leakage will provide an entrance for a drainage tube to penetrate the esophagus, and a tube through the anastomotic stoma will hinder the healing. Since cervical drainage is a common therapy for cervical anastomotic leakage, the drainage tube may not be eliminated and stay in the esophagus, keeping the anastomotic stoma from healing. This situation may be neglected in clinical practice if lack of experience. Furthermore, the penetrating of the drainage tube may be the reason for anastomotic leakage, though lack of enough evidence to prove it, which requires further studies.

After reviewing the presented cases, we summarized some key points to help diagnose as follows:

1. Duration of leakage.

The median length of duration in hospital is 3 weeks approximately and seldom exceeds 40 days as reported in some studies (13–15). If the disease course of a patient with anastomotic leakage is significantly longer than usual, clinicians should be alert and consider the possibility of the drainage tube penetrating the anastomotic stoma.

2. Drainage fluids amounts and characteristics.

The volume of drainage fluids is usually lower than 50 ml/24 h since the second postoperative day and the difference in amount between patients with or without leakage is not significant (10). However, if the drainage tube penetrates the anastomotic stoma and goes deep into the stomach, the gastric juice may be drained thus increasing the daily volume of drainage fluids significantly (up to 800 ml in the presented case 1) and turning the clear, hematic, light red, or dark red fluids into yellow-green or brown mucus due to the mixing into of gastric juice. If the amounts and characteristics of drainage fluids alter and the color turns to yellow-green with an increasing amount of gas coming out, the possibility of the drainage tube penetrating the anastomotic stoma should be considered. A pH test of drainage fluids may help diagnose because of gastric juice acidity.

3. Radiographic manifestations.

Esophagography and CT are common techniques for anastomotic leakage diagnosis. CT is superior in sensitivity while esophagography is more specific and cost-effective for anastomotic leakage diagnosis (16, 17). If a drainage tube penetrates the anastomotic stoma, the contrast agent will be drained and presented in images when carrying out esophagography. However, the contrast agent may also be drained when anastomotic leakage occurs without the tube penetrating. Under such situations, esophagography is capable to diagnose anastomotic leakage but not enough to locate the drainage tube, hence the CT is necessary for locating the drainage tube. Dynamic observation of the location of the neck drainage tube in different slices of CT could show clearly that the neck drainage tube penetrates the esophageal cavity.

4. Endoscopy.

Endoscopic evaluation of the conduit and anastomosis after esophagectomy has shown superiority in technical feasibility, sensitivity, and specificity (18). Effective intervention such as endoscopic vacuum therapy, endoscopic clip placement, injection of fibrin glue, and endoscopic insertion of transluminal drainages is available simultaneously under endoscopy (19).

Since drainage tube is important for patients who received McKeown esophagectomy but the drainage tube may hinder the recovery, the management of drainage tube is essential. A proper timing for tube removal is important. However, the timing for removing the cervical drainage tube is still controversial. There are multiple standards for cervical drainage tube removal such as removing on the first postoperative day except for patients with active bleeding or removing when the daily output amount reduces to 20 ml or less (10, 20). Different indications could be expected to result in a huge difference in the drainage tube placement time. Longer drainage tube placement time may increase the risk of tube penetrating while a shorter time may cause inadequate drainage and delay the healing (21). In fact, we have encountered more similar cases in our clinical practice. Learning from those cases, we decide to set a tube removal timing indicator as a less drainages than 20 ml in our following clinical practice based on medical literatures and our clinical experience, and we noticed possible better recovery of patients with anastomotic leakage, indicating that a proper cervical drainage tube removal time is conducive to the healing of anastomotic leakage, which required further retrospective studies or clinical trials to verify. To reduce the possibility of the position change of the cervical drainage tube, the tube should be fixed stably. Position markers could be labeled to help clinicians to notice that the position of drainage tube has changed so that intervention could start as soon as possible. The size of drainage tubes is another noteworthy factor. The size of drainage tubes ranging from 15 to 18 Ch/Fr is recommended (10). The material of drainage tubes should also be taken into consideration. A softer tube may reduce the risk of penetrating.

## Conclusion

We presented two cases about a rare cause of long-term nonunion of esophagogastric anastomosis in neck, which is the drainage tube penetrating anastomosis, described the disease progression, clinical diagnosis, and management, summarized the diagnosis key points, and suggested the solutions, to draw clinicians’ attention to this rare but noteworthy situation.

## Data Availability

The original contributions presented in the study are included in the article/**Supplementary Material**, further inquiries can be directed to the corresponding authors.
