# Supplementary material for: Case report: Drainage tube penetrating anastomosis as a rare cause for long-term nonunion of esophagogastric anastomosis in neck
Source: Front Surg. 2023 Feb 22;10:1140839. doi: 10.3389/fsurg.2023.1140839 (PMC9992177; doi:10.3389/fsurg.2023.1140839)
Supplement: Supplementary file 1 [file Datasheet1.pdf]

CARE
case report guidelines

CARE Checklist of information to include when writing a case report

(cc) BY-NC-ND

Topic	Item	Checklist item description	Reported on Line
Title	1	The diagnosis or intervention of primary focus followed by the words "case report"	1-2
Key Words	2	2 to 5 key words that identify diagnoses or interventions in this case report, including "case report"	27-28
Abstract (no references)	3a	Introduction: What is unique about this case and what does it add to the scientific literature?	13-15
	3b	Main symptoms and/or important clinical findings	16-20
	3c	The main diagnoses, therapeutic interventions, and outcomes	18-21
	3d	Conclusion—What is the main "take-away" lesson(s) from this case?	23-26
Introduction	4	One or two paragraphs summarizing why this case is unique (**may include references**)	39-42
Patient Information	5a	De-identified patient specific information.	45-46; 81-82
	5b	Primary concerns and symptoms of the patient.	47-48; 83-84
	5c	Medical, family, and psycho-social history including relevant genetic information	46-53; 82-87
	5d	Relevant past interventions with outcomes	53-57; 53-57
Clinical Findings	6	Describe significant physical examination (PE) and important clinical findings.	59-79; 96-110
Timeline	7	Historical and current information from this episode of care organized as a timeline	59-79; 96-110
Diagnostic Assessment	8a	Diagnostic testing (such as PE, laboratory testing, imaging, surveys).	70-72; 99-101
	8b	Diagnostic challenges (such as access to testing, financial, or cultural)	96-99
	8c	Diagnosis (including other diagnoses considered)	59-60
	8d	Prognosis (such as staging in oncology) where applicable	51-52; 56-57; 86-87; 91-94
Therapeutic Intervention	9a	Types of therapeutic intervention (such as pharmacologic, surgical, preventive, self-care)	60-61; 97
	9b	Administration of therapeutic intervention (such as dosage, strength, duration)	60-61; 97
	9c	Changes in therapeutic intervention (with rationale)	73; 105
Follow-up and Outcomes	10a	Clinician and patient-assessed outcomes (if available)	78-79; 109-110
	10b	Important follow-up diagnostic and other test results	75-76; 106-107
	10c	Intervention adherence and tolerability (How was this assessed?)	not available
	10d	Adverse and unanticipated events	not avaliable
Discussion	11a	A scientific discussion of the strengths AND limitations associated with this case report	113-200
	11b	Discussion of the relevant medical literature **with references**.	113-200
	11c	The scientific rationale for any conclusions (including assessment of possible causes)	144-200
	11d	The primary "take-away" lessons of this case report (without references) in a one paragraph conclusion	202-206
Patient Perspective	12	The patient should share their perspective in one to two paragraphs on the treatment(s) they received	84-88; 105-106
Informed Consent	13	Did the patient give informed consent? Please provide if requested	**Yes** ☑ **No** ☐
